# Unsupervised machine learning identifies distinct phenotypes in cardiac complications of pediatric patients treated with anthracyclines

**DOI:** 10.1186/s40959-024-00276-4

**Published:** 2024-10-28

**Authors:** Xander Jacquemyn, Bhargava K. Chinni, Benjamin T. Barnes, Sruti Rao, Shelby Kutty, Cedric Manlhiot

**Affiliations:** 1 Department of Pediatrics, The Blalock-Taussig-Thomas Pediatric and Congenital Heart Center, Johns Hopkins School of Medicine, Johns Hopkins University, Johns Hopkins Hospital, 600 N. Wolfe Street, 1389 Blalock, Baltimore, MD 21287 USA; 2 Department of Cardiovascular Sciences, KU Leuven & Congenital and Structural Cardiology, UZ Leuven, Leuven, Belgium

**Keywords:** Machine learning, Anthracycline, Cancer therapy–related cardiac dysfunction, Cardiotoxicity, Echocardiography

## Abstract

**Background:**

Anthracyclines are essential in pediatric cancer treatment, but patients are at risk cancer therapy-related cardiac dysfunction (CTRCD). Standardized definitions by the International Cardio-Oncology Society (IC-OS) aim to enhance precision in risk assessment.

**Objectives:**

Categorize distinct phenotypes among pediatric patients undergoing anthracycline chemotherapy using unsupervised machine learning.

**Methods:**

Pediatric cancer patients undergoing anthracycline chemotherapy at our institution were retrospectively included. Clinical and echocardiographic data at baseline, along with follow-up data, were collected from patient records. Unsupervised machine learning was performed, involving dimensionality reduction using principal component analysis and K-means clustering to identify different phenotypic clusters. Identified phenogroups were analyzed for associations with CTRCD, defined following contemporary IC-OS definitions, and hypertensive response.

**Results:**

A total of 187 patients (63.1% male, median age 15.5 years [10.4–18.7]) were included and received anthracycline chemotherapy with a median treatment duration of 0.66 years [0.35–1.92]. Median follow-up duration was 2.78 years [1.31–4.21]. Four phenogroups were identified with following distribution: Cluster 0 (32.6%, *n* = 61), Cluster 1 (13.9%, *n* = 26), Cluster 2 (24.6%, *n* = 46), and Cluster 3 (28.9%, *n* = 54). Cluster 0 showed the highest risk of moderate CTRCD (HR: 3.10 [95% CI: 1.18–8.16], *P* = 0.022) compared to other clusters. Cluster 3 demonstrated a protective effect against hypertensive response (HR: 0.30 [95% CI: 0.13– 0.67], *P* = 0.003) after excluding baseline hypertensive patients. Longitudinal assessments revealed differences in global longitudinal strain and systolic blood pressure among phenogroups.

**Conclusions:**

Unsupervised machine learning identified distinct phenogroups among pediatric cancer patients undergoing anthracycline chemotherapy, offering potential for personalized risk assessment.

**Supplementary Information:**

The online version contains supplementary material available at 10.1186/s40959-024-00276-4.

## Introduction

Anthracyclines are crucial agents for treating various cancers in children and adolescents. Despite an 85% survival rate [1], long-term survivors face cancer therapy-related risks, notably cardiac dysfunction (CTRCD), ranging from asymptomatic reduction in left ventricular ejection fraction (LVEF) to overt heart failure (HF) [2]. The Children’s Oncology Group (COG) reported that around 12% of pediatric acute myeloid leukemia patients experienced cardiotoxicity [3]. To address varied estimates of cardiotoxicity risk, the International Cardio-Oncology Society (IC-OS) recently issued a consensus statement to standardize definitions, aiming to improve communication and enhance research on clinical outcomes [4]. Increased awareness and assessment of cardiotoxicity using both LVEF and global longitudinal strain (GLS) could guide more effective cardioprotective measures to achieve improved long-term outcomes [5]. In contemporary clinical practice, the risk stratification of these patients relies on established methods that use predefined criteria and known risk factors identified in prior observational or randomized studies [6]. These conventional approaches typically categorize patients into broad risk groups based on factors like age, gender, diagnosis, and comorbidities [6]. Unsupervised machine learning approaches provide a unique opportunity to improve risk stratification and patient phenotyping by examining patterns and structures within a given population [7]. For example, a previous machine learning model developed for prediction of cardiotoxicity, combining genetic and clinical factors, outperformed models relying solely on clinical variables, while exhibiting high specificity and a low misclassification rate [8]. Our hypothesis was based upon utilizing baseline clinical variables and the evolution of cardiac function and blood pressure parameters to discern distinct phenotypes of pediatric patients undergoing anthracycline chemotherapy. As these variables are readily available in clinical practice, this phenogrouping could potentially lead to a more detailed comprehension of the diversity in disease progression over time, and uncover potential avenues for patient-tailored treatment strategies.

## Methods

### Study population

We retrospectively identified patients with cancer who underwent anthracycline chemotherapy (specifically doxorubicin, epirubicin, daunorubicin, idarubicin, or mitoxantrone) as part of their treatment at our institution from January 2013 to September 2021. Patients receiving other medications with potential cardiotoxic effects were excluded. The cumulative total dose of anthracyclines was converted to a doxorubicin equivalent using evidence-based equivalence ratios [9]. Throughout their cancer treatment and subsequent post-chemotherapy follow-up, these patients underwent cardiac assessment using 2-dimensional echocardiography at clinically indicated intervals, as determined by their treating oncologist. To be eligible for the study, patients were required to have undergone a baseline assessment at maximum 25 days prior to starting chemotherapy, have undergone a minimum of 2 total echocardiography assessments, and have longitudinal follow-up at our institution. Exclusion criteria consisted of inadequate image quality for myocardial deformation analysis, any level of valvular stenosis, valvular regurgitation, or a history of prior heart failure. The study was approved by the Johns Hopkins Medicine institutional review board with waiver of informed consent due to the retrospective nature of the study. Sex- and age-standardized weight, height, and BMI metrics were obtained from Centers for Disease Control and Prevention (CDC) growth charts for the United States [10]. Blood pressure (BP) was measured using an automated BP cuff in the sitting position, prior to performing an echocardiogram. An appropriately sized cuff was chosen and BP was measured in the right arm, unless otherwise indicated in the standard measurement guidelines [11]. Age- and height adjusted Z-scores for BP were obtained from the Boston Children’s Hospital Z-score calculator [12]. 

### Echocardiography measurements and analysis

All patients underwent a comprehensive transthoracic echocardiographic assessment as part of their routine chemotherapy surveillance. All echocardiograms were performed using a commercially available ultrasound system (Vivid 9, General Electric Medical Systems, Horten, Norway). Standard imaging windows and measurements were made according to the American Society of Echocardiography guidelines following a standardized protocol [13]. Left ventricular ejection fraction (LVEF) was calculated using Simpson’s biplane measurement in the apical four- and two-chamber views. Left ventricular fractional shortening (LVFS) was obtained from M-mode imaging in the parasternal long-axis view. Global longitudinal strain (GLS) was measured using speckle tracking echocardiography (STE) in the apical four-chamber, two-chamber, and three-chamber views. Tricuspid annular plane systolic excursion (TAPSE) was obtained from m-mode imaging in the apical four-chamber view.

### Study outcomes

The primary outcome consisted of asymptomatic cancer therapy-related cardiac dysfunction (CTRCD) classified into categories following recommendations by the International Cardio-Oncology Society 2021 Consensus statement: mild CTRCD indicates a LVEF of ≥ 50% along with a new relative decline in GLS > 15% from baseline and/or a new elevation in cardiac biomarkers; moderate CTRCD encompasses an absolute LVEF reduction of ≥ 10% resulting in an LVEF of 40–49%, or a reduction of < 10% points to an LVEF between 40 and 49%, coupled with a new relative decline in GLS > 15% from baseline and/or a new elevation in cardiac biomarkers; while severe CTRCD denotes a new LVEF reduction to < 40%. The secondary outcome consisted of exaggerated hypertensive response to the chemotherapy regimen, and was defined as SBP increase > 20 mm Hg or mean arterial pressure (MAP) increase > 15 mmHg from baseline [4, 14]. 

### Data processing

Missing data was evaluated, and features exceeding 40% missingness were omitted from the analysis. Among the 47 features considered for analysis (Supplemental Table 1), 5 had missingness between 20% and 40%. To handle missing data, individual prediction models were created using the XGBoost regression algorithm, was trained on a complete dataset using a 75/25 train/test split [15]. The Boruta feature selection algorithm was applied to the training set to retain the features that had statistical significance in predicting the target variables [16], and using this subset of features a Bayesian hyperparameter tuning process was employed to train the XGBoost model, determine the optimal training parameters, and evaluate the model performance [17, 18]. Following model development and evaluation, missing values were estimated for those 5 target variables. The remaining variables with less than 20% missingness were imputed using an iterative imputer, taking into consideration the similarity among patients to estimate the missing values [19]. For each patient, to assess phenotypic response to therapy, change in the following variables was measured: LVESV, LVEDV, LVEF, GLS, SBP and DBP. This value of each variable at timepoint T was quantified using a quadratic function according to the following formula:

$$\eqalign{& ResponseTherap{y_T} = Variabl{e_{T0}} + Slope*Time + \cr & \,\,\,\,\,\,\,\,\,\,\,\,\,\,\,\,\,\,\,\,\,\,\,\,\,\,\,\,\,\,\,\,\,\,\,\,\,\,\,\,\,\,\,\,\,\,Quadratic\,Coefficient*Time \cr}$$

### Unsupervised machine learning analysis

Following imputation, the dataset was standardized prior to principal component analysis (PCA) [20]. Unsupervised learning analyzes unlabeled datasets to uncover inherent patterns and structures without relying on dependent variables, unlike supervised learning methods which require a “ground truth”, typically a class or an outcome [7]. Within the group of unsupervised machine learning, techniques such as PCA are often employed for dimensionality reduction, simplifying data by reducing its dimensions while preserving variability. PCA generates principal components (PC), which are linear combinations of the original variables, capturing the most significant variations in the data [7]. Clustering methods, including k-means and hierarchical clustering, then group data points sharing similarities into distinct clusters; for instance, k-means allocates data points to clusters based on their proximity to the cluster centroids [7]. In our study, PCA was applied prior to clustering to reduce data dimensionality (Supplemental Table 2). Subsequently, 5 PCs that accounted for 47.8% of the variance in the data were selected due to a significant “elbow” at the 5th PC in the explained variance ratio (Supplemental Fig. 1). These selected PCs were then used as input for unsupervised cluster analysis (Supplemental Table 3). After dimensionality reduction using PCA, we used K-means clustering algorithm to segment patients into distinct phenogroups based on their similarity, which was optimized using a grid search by varying the number of clusters [21]. We implemented an iterative process to determine the optimal configuration for K-means clustering, maximizing the silhouette score as the evaluation metric, while minimizing the within-cluster sum of squares (WCSS) [22]. The optimal configuration segmented the patients into four clusters, each accounting for 32.6%, 13.9%, 24.6% and 28.9% of the population. Cluster labels were obtained from the K-means clustering for each patient and phenomap analysis was implemented to identify the set of similar patient characteristics across each cluster and the set of characteristics that differentiate between the clusters.

### Statistical analysis

Normality of the distribution of continuous variables was tested using the Shapiro–Wilk test. Continuous variables are expressed as mean with standard deviation or median with interquartile range (IQR), as appropriate. Categorical variables are expressed as counts and relative frequency (%). Time-to-event data were plotted using the Kaplan–Meier method. To assess the relationship between clusters and CTRCD, we employed Cox regression models. A linear mixed-effects model using cubic polynomials was used to describe the relationship between follow-up variables across clusters. To evaluate whether cluster group membership had a significant impact on these variables, a likelihood ratio test (LRT) was used. The LRT compares the full model, including cluster groups and interactions, to a reduced model that excludes these terms. A 2-tailed p-value < 0.05 was considered statistically significant. All analyses were completed with R Statistical Software (version 4.1.1, Foundation for Statistical Computing, Vienna, Austria) and Python (version 3.11.3, Python Software Foundation).

## Results

### Study population

A total of 187 patients (median age 15.5 [IQR 10.4, 18.7] years, range 4.6 months to 24.7 years, 118 males [63.1%]) underwent anthracycline chemotherapy for the treatment of primary malignancy. Clinical characteristics of the study cohort are summarized in Table 1, along with the percentage of missingness for that variable. The chemotherapeutic regimen had a median duration of 0.66 (IQR 0.35, 1.92) years accounting for a median total cumulative anthracycline exposure of 200 (IQR 135, 343) mg/m^2^.

**Table 1:** Characteristics of the entire chemotherapy cohort

Characteristic	Missing (%)	Total (*N* = 187)
Age, years	0 (0.0)	15.5 [10.4, 18.7]
Male, n (%)	0 (0.0)	118 (63.1)
Height, cm	1 (0.5)	163 [135, 175]
BMI, kg/m^2^	3 (1.6)	21.1 [17.0, 25.5]
Hypertension, n (%)	2 (1.1)	32 (17.1)
Diagnosis		
Lymphoma, n (%)	0 (0.0)	50 (26.7)
Sarcoma, n (%)	0 (0.0)	49 (26.2)
AML, n (%)	0 (0.0)	14 (7.5)
ALL, n (%)	0 (0.0)	36 (19.3)
Blastoma, n (%)	0 (0.0)	8 (4.3)
Wilms tumor, n (%)	0 (0.0)	10 (5.4)
Other, n (%)	0 (0.0)	20 (10.7)
Baseline cardiac medications, n (%)	0 (0.0)	0 (0.0)
Duration of chemotherapy, years	3 (1.6)	0.66 [0.35, 1.92]
Cumulative anthracycline exposure^a^, mg/m^2^	3 (1.6)	200 [135, 343]
Duration of follow-up, years	0 (0.0)	2.78 [1.31, 4.21]

LEGEND Normally distributed variables were presented as mean ± standard deviation while non-normally distributed variables were presented as median (interquartile range). Categorical variables are expressed as frequency (percentage). ABBREVIATIONS: ALL, acute lymphoblastic leukemia; AML, acute myeloid leukemia; BMI, body mass index

### Exploration of the low-dimensional output space

**Fig. 1:**
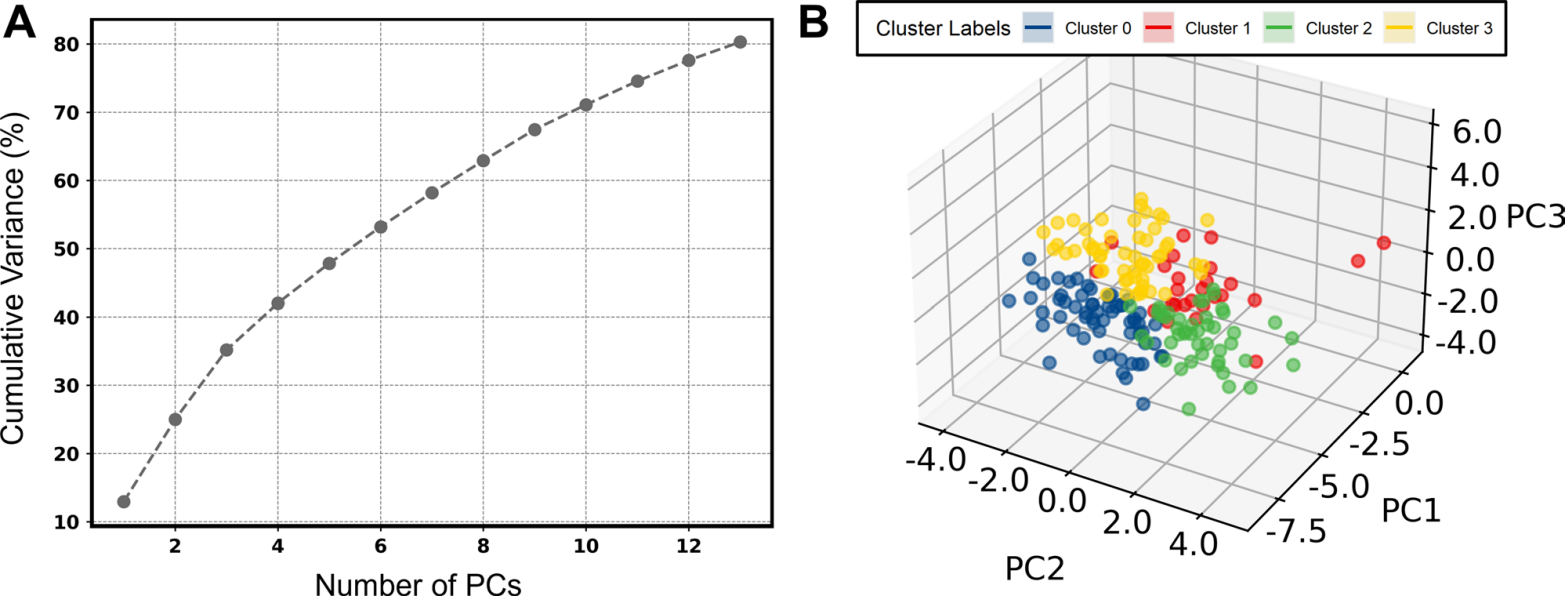
Principal components and clustering of the low dimensional output space. Legend: Percentage of explained cumulative variance by the principal components (**A**) and positioning of the included patients in the 3 first principal components of the output space obtained after dimensionality reduction, colored according to the assigned distinct cluster phenotypes obtained from the K-means clustering algorithm (**B**). Abbreviations: PC: principal component

**Fig. 2:**
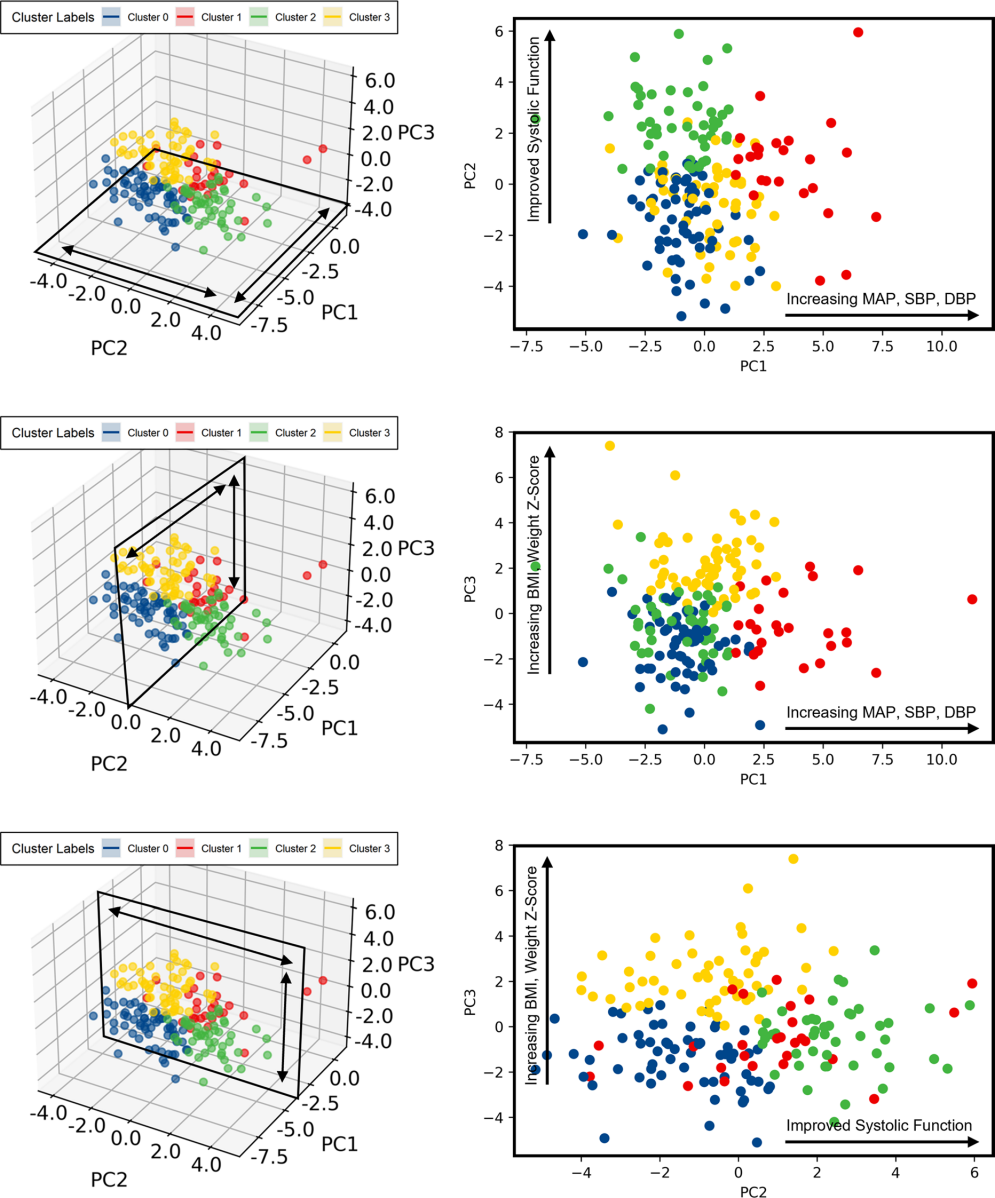
Exploration of the low dimensional output space. Legend: Exploration of clusters according to the first 3 principal components in each direction. Abbreviations: BMI, body mass index; DBP, diastolic blood pressure; MAP, mean arterial pressure; PC: principal component; SBP, systolic blood pressure

In total, 80.3% of the variance could be explained by 14 PCs, however, the PCA analysis consisted of 5 PCs that accounted for 47.8% of the variance (Fig. 1A). Unsupervised k-means clustering identified four distinct clusters, which corresponded to the quadrants identified by PCA (Fig. 1B). Exploration of the first 3 PCs (explaining 35.4% of the variance) revealed that (1) PC1 seems to represent a combination of various blood pressure metrics at baseline (MAP Z-score, DBP Z-score and SBP Z-score) as well as their longitudinal changes in response to therapy, (2) PC2 was most influenced by features that capture variations related to different cardiac functional metrics (LVEF, LVFS, GLS), and lastly (3) PC3 was most represented by variations related strongly to anthropometric parameters (BMI Z-score, weight- and height-for-age Z-scores, BSA) (Fig. 2) (Supplemental Fig. 2).

### Unsupervised machine learning revealed four distinct phenogroups

Patient characteristics according to identified phenogroups are presented in Table 2. Cluster 0 (*n* = 61 patients) consisted of adolescents (median 17.1 [IQR 12.0, 19.4] years at baseline), with lymphoma being the most common diagnosis (36.1%). Patients in Cluster 0 demonstrated already high levels of abnormal GLS at baseline (62.3%). Cluster 1 (*n* = 26 patients) consisted of younger children (median 5.8 [IQR 2.28, 12.4] years at baseline), with acute lymphoblastic leukemia (ALL) being the most common diagnosis (30.8%). Characteristic about this cluster is the high level of elevated BP (92.3%), and hypertension (76.9% Stage 1, and 34.6% Stage 2), demonstrating the largest increases in DBP Z-score. Furthermore, patients within this cluster were frequently underweight (76.9%). Cluster 2 (*n* = 46 patients) was characterized by relatively preserved LVEF and GLS compared to the other groups. Furthermore, this cluster had the highest frequency of female patients (47.8%) and were very frequently underweight (60.9%). Cluster 3 (*n* = 54 patients) can be classified as having more obesity (55.6% overweight, and 22.2% obese) and systolic hypertension (14.8%), potentially indicating a metabolic syndrome-like profile.

**Table 2:** Baseline characteristics according to identified phenotypes

Characteristic	Cluster 0 (*N* = 61)	Cluster 1 (*N* = 26)	Cluster 2 (*N* = 46)	Cluster 3 (*N* = 54)	Overall *p*-value
Demographics					
Age, years	17.1 [12.0, 19.4]	5.80 [2.28, 12.4]	13.4 [9.96, 17.1]	16.7 [15.0, 18.8]	< 0.001^*†¶§#^
Male, n (%)	42 (68.9)	16 (61.5)	24 (52.2)	36 (66.7)	0.32
Diagnosis					< 0.001^*†¶§#^
Lymphoma, n (%)	22 (36.1)	3 (11.5)	10 (21.7)	15 (27.8)	
Sarcoma, n (%)	17 (27.9)	3 (11.5)	13 (28.3)	16 (29.6)	
AML, n (%)	8 (13.1)	1 (3.9)	0 (0.0)	5 (9.3)	
ALL, n (%)	8 (13.1)	8 (30.8)	8 (17.4)	12 (22.2)	
Blastoma, n (%)	0 (0.00)	4 (15.4)	4 (8.7)	0 (0.0)	
Wilms tumor, n (%)	2 (3.3)	6 (23.1)	2 (4.4)	0 (0.0)	
Other, n (%)	4 (6.6)	1 (3.9)	9 (19.6)	6 (11.1)	
Clinical assessment					
BMI Z-score	0.51 [-0.34, 0.99]	-0.44 [-0.97, 0.52]	-0.67 [-1.27, 0.38]	1.28 [0.83, 1.76]	< 0.001^*†‡§#^
Height Z-score	0.34 [-0.50, 1.00]	0.81 [-0.22, 1.43]	-0.30 [-1.34, 0.26]	0.49 [-0.24, 1.12]	< 0.001^†¶#^
Weight Z-score	0.59 [-0.24, 1.18]	0.00 [-0.63, 0.54]	-0.63 [-1.25, -0.14]	1.55 [0.81, 2.11]	< 0.001^†‡¶§#^
Systolic BP Z-score	0.17 (0.99)	1.79 (0.81)	-0.06 (1.04)	1.50 (0.97)	< 0.001^*‡¶#^
Diastolic BP Z-score	0.27 [-0.22, 0.88]	2.33 [1.85, 3.23]	0.42 [-0.19, 0.65]	0.94 [0.34, 1.77]	< 0.001^*‡¶§#^
MAP Z-score	0.14 (0.69)	2.08 (0.89)	-0.08 (0.80)	1.08 (0.86)	< 0.001^*‡¶§#^
HTN classification					
Elevated blood pressure, n (%)	11 (18.0)	24 (92.3)	9 (19.6)	19 (35.2)	< 0.001^*¶§^
Stage 1 HTN, n (%)	1 (1.7)	20 (76.9)	4 (8.7)	7 (13.0)	< 0.001^*‡¶§^
Stage 2 HTN, n (%)	0 (0.0)	9 (34.6)	0 (0.0)	1 (1.85)	< 0.001^*¶§^
Baseline echocardiography assessment					
LVESV, ml	28.1 [21.9, 37.7]	14.5 [9.61, 24.4]	16.8 [11.7, 21.6]	25.8 [21.9, 31.5]	< 0.001^*†§#^
LVEDV, ml	77.4 [56.9, 95.6]	39.7 [28.1, 57.0]	50.2 [37.8, 67.0]	81.6 [68.5, 96.1]	< 0.001^*†§#^
LVESVi, ml/m^2^	19.3 [15.3, 21.7]	15.3 [12.8, 17.6]	13.3 [11.0, 15.3]	13.7 [12.0, 16.2]	< 0.001^*†‡^
LVEDVi, ml/m^2^	48.0 [41.4, 55.5]	40.3 [36.5, 44.2]	41.2 [33.2, 47.6]	42.1 [37.0, 49.5]	0.002^*†‡^
LVFS, %	33.6 [31.4, 36.5]	35.7 [32.9, 38.9]	39.6 [37.4, 42.6]	40.0 [36.9, 42.9]	< 0.001^*†‡¶§^
LVEF, %	61.2 (3.5)	63.7 (5.2)	67.7 (4.0)	66.6 (4.7)	< 0.001^*†‡¶§^
GLS, %	-18.65 (2.7)	-21.48 (2.6)	-22.37 (2.2)	-20.54 (2.9)	< 0.001^*†‡#^
Abnormal GLS, n (%)	38 (62.3)	7 (26.9)	7 (15.2)	18 (33.3)	< 0.001^*†‡^
TAPSE, cm	2.31 (0.5)	2.06 (0.3)	2.19 (0.4)	2.60 (0.4)	< 0.001^‡§#^
Follow-up					
Duration of chemotherapy, years	0.51 [0.34, 0.83]	1.25 [0.52, 2.10]	0.64 [0.36, 2.28]	0.72 [0.33, 1.88]	0.080^*^
Cumulative anthracycline exposure^a^, mg/m^2^	245 [168, 322]	148 [80.3, 179]	207 [122, 360]	200 [135, 371]	0.001^*¶§^
Cumulative anthracycline exposure > 250 mg/m^2^, n (%)	21 (34.4)	3 (11.5)	18 (39.1)	20 (37.0)	0.099
Duration of follow-up, years	3.02 [1.74, 4.82]	1.48 [0.60, 2.57]	2.80 [1.65, 3.61]	2.79 [1.49, 4.42]	0.021^*¶§^
Cardiac medications during follow-up					
Angiotensin-converting enzyme inhibitors, n (%)	17 (27.9)	1 (3.9)	1 (2.2)	9 (16.7)	0.001^*†#^
Beta-blockers, n (%)	5 (8.2)	1 (3.9)	1 (2.2)	1 (1.9)	0.36

LEGEND Normally distributed variables were presented as mean ± standard deviation while non-normally distributed variables were presented as median (interquartile range). Categorical variables are expressed as frequency (percentage). Pairwise comparisons were adjusted for multiple testing using the Tukey correction method and Benjamini-Hochberg method as appropriate. * Significant *p*-value between Cluster 0 and Cluster 1, † Significant *p*-value between Cluster 0 and Cluster 2, ‡ Significant *p*-value between Cluster 0 and Cluster 3, ¶ Significant *p*-value between Cluster 1 and Cluster 2, § Significant *p*-value between Cluster 1 and Cluster 3, # Significant *p*-value between Cluster 2 and Cluster 3. ^a^Cumulative lifetime dose expressed as doxorubicin equivalent. ABBREVIATIONS: ALL, acute lymphoblastic leukemia; AML, acute myeloid leukemia; BMI, body mass index; GLS, global longitudinal strain; HTN, hypertension; LVEF, left ventricular ejection fraction, LVEDV, LV end diastolic volume, LVESV, LV end systolic volume, LVFS, left ventricular fractional shortening; LVEDVi, LVEDV indexed to BSA; LVESVi, LVESV indexed to BSA; MAP, mean arterial pressure; TAPSE, tricuspid annular plane systolic excursion

**Fig. 3:**
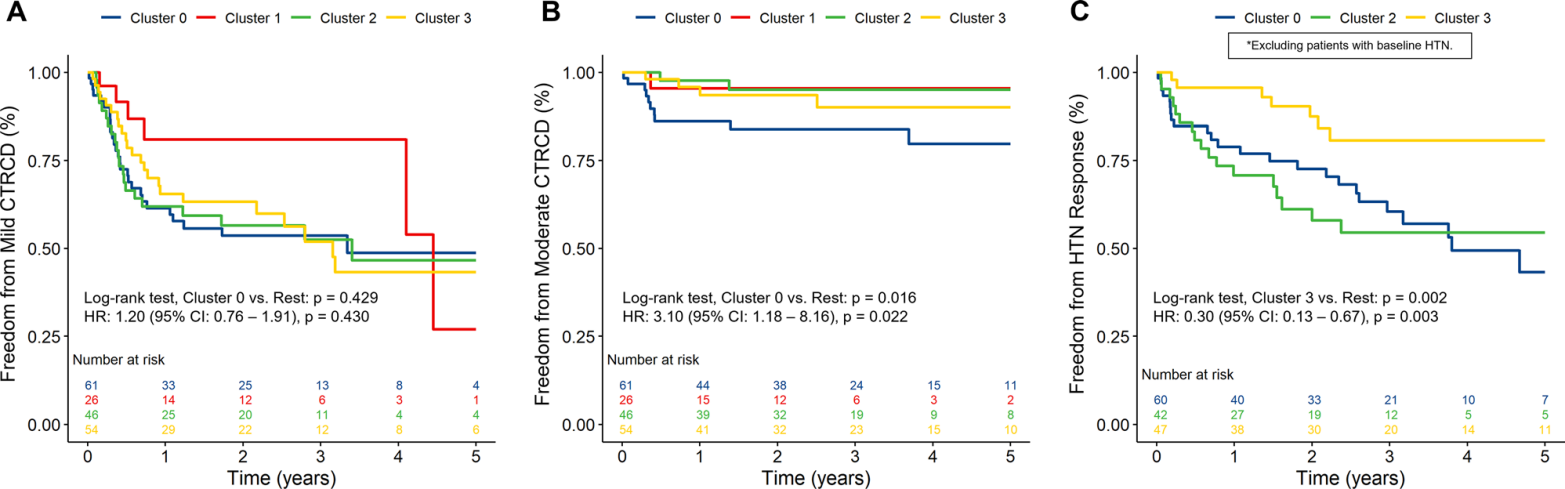
Freedom from cancer therapy-related cardiac dysfunction and hypertensive response. Legend: Kaplan–Meier curves for the primary outcomes (**A**) mild CTRCD, (**B**) moderate CTRCD and (**C**) hypertensive response stratified according to identified phenotypes. *Patients with baseline hypertension (*N* = 38 patients) were excluded from the analysis of hypertensive response. Abbreviations: CI, confidence interval; CTRCD, cancer therapy-related cardiac dysfunction; HR, hazard ratio; HTN, hypertension

During longitudinal follow-up (median of 2.8 [IQR 1.3–4.2] years), cumulative anthracycline exposure varied between groups, with Cluster 0 receiving the highest doses on average (245 [IQR 168, 322] mg/m^2^). In contrast, Cluster 1 had the shortest duration of anthracycline chemotherapy and received the lowest doses. The early event rates for mild CTRCD, moderate CTRCD and hypertensive response at 6 months and 1 year follow-up for patients with complete follow-up is shown in Table 3. During longer follow-up, 81 patients (43.3%) experienced mild CTRCD, 17 patients (9.1%) experienced moderate CTRCD and 3 patients (1.6%) experienced severe CTRCD. Kaplan–Meier analysis of long-term CTRCD stratified by phenogroups identified on k-means clustering is shown in Fig. 3A-B. Cluster 0 demonstrated the highest risk for moderate CTRCD compared to the other phenotypes (HR: 3.10 [95% CI: 1.18–8.16], *P* = 0.022). In line with this observation, Cluster 0 also received angiotensin-converting enzyme inhibitors more frequently (27.9%) compared to the other phenotypes (8.7%).

**Table 3:** Early events stratified according to identified phenotype with complete follow-up

Outcomes	Event at 6 months (%)	Relative Risk (95% CI)	Event at 1 year (%)	Relative Risk (95% CI)
Mild CTRCD	41 / 168 (24.4)		48 / 149 (32.2)	
Cluster 0	15 / 55 (27.3)	Reference	17 / 50 (34.0)	Reference
Cluster 1	2 / 21 (9.5)	0.35 (0.09–1.40)	2 / 16 (12.5)	0.37 (0.10–1.42)
Cluster 2	15 / 44 (34.1)	1.25 (0.69–2.27)	15 / 40 (37.5)	1.10 (0.63–1.92)
Cluster 3	9 / 48 (18.8)	0.69 (0.33–1.43)	14 / 43 (32.6)	0.96 (0.54–1.71)
Moderate CTRCD	11 / 168 (6.5)		10 / 149 (6.7)	
Cluster 0	8 / 55 (14.5)	Reference	6 / 50 (12.0)	Reference
Cluster 1	1 / 21 (4.8)	0.33 (0.04–2.46)	1 / 16 (6.3)	0.52 (0.07–4.01)
Cluster 2	1 / 44 (2.3)	0.16 (0.02–1.20)	1 / 40 (2.5)	0.21 (0.03–1.66)
Cluster 3	1 / 48 (2.1)	0.14 (0.02–1.10)	2 / 43 (4.7)	0.39 (0.08–1.82)
Hypertensive response	18 / 168 (10.7)		20 / 149 (13.4)	
Cluster 0	9 / 55 (16.4)	Reference	9 / 50 (18.0)	Reference
Cluster 1	0 / 21 (0.0)	0.13 (0.01–2.20)	0 / 16 (0.0)	0.15 (0.01–2.57)
Cluster 2	7 / 44 (15.9)	0.97 (0.39–2.40)	9 / 40 (22.5)	1.25 (0.55–2.85)
Cluster 3	2 / 48 (4.2)	0.25 (0.06–1.12)	2 / 43 (4.7)	0.26 (0.06–1.13)

ABBREVIATIONS CTRCD, cancer therapy-related cardiac dysfunction

**Fig. 4:**
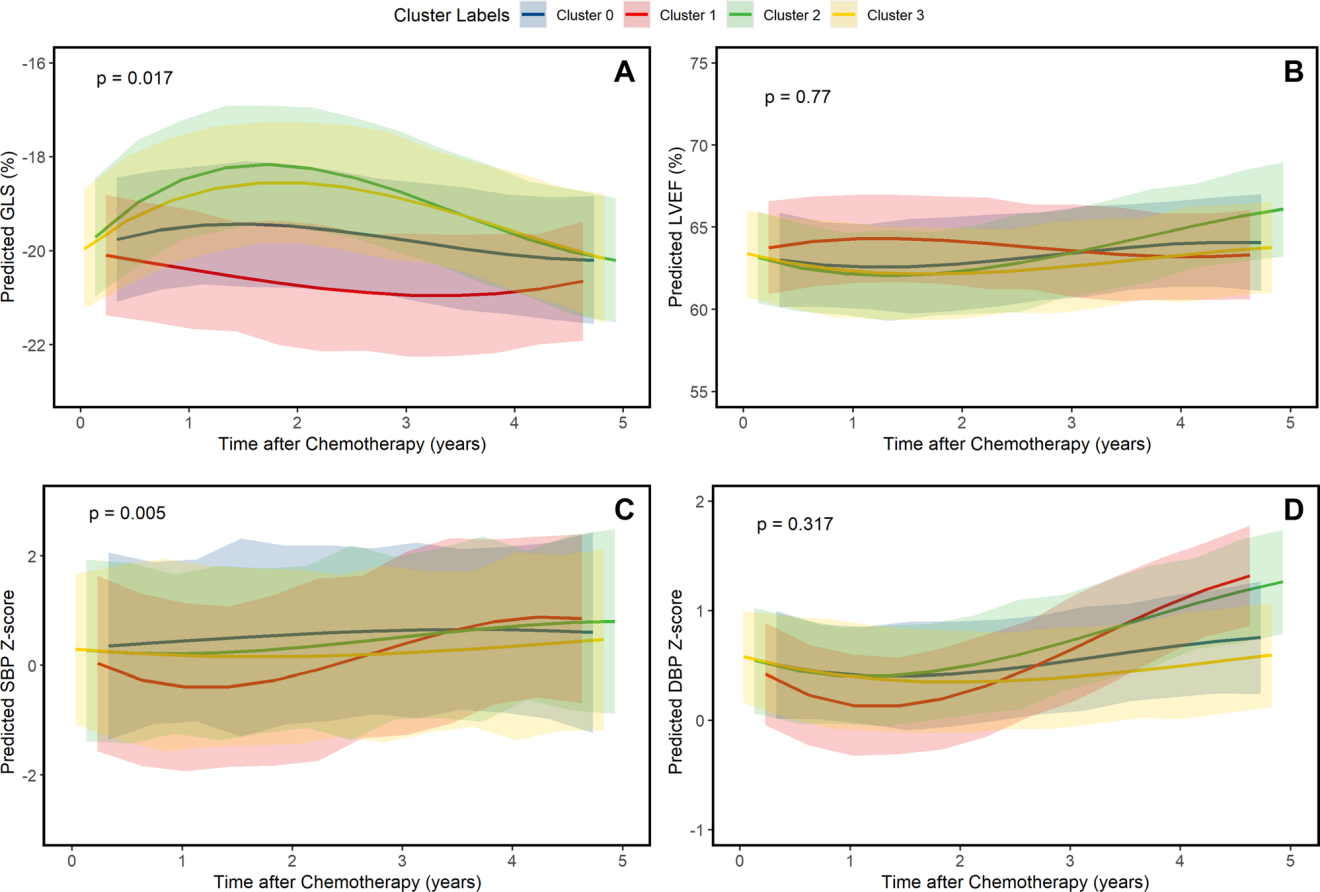
Differences in blood pressure and systolic function. Legend: Echocardiographic and clinical trajectories stratified according to identified phenotypes. Abbreviations: DBP, diastolic blood pressure; GLS, global longitudinal strain; LVEF, left ventricular ejection fraction; SBP, systolic blood pressure

From the 17 total patients with moderate CTRCD, only 5 patients (29.4%) recovered completely. Recovery varied when stratified by phenotypes identified on k-means clustering (4 recoveries in Cluster 0 [40.0%], 1 recovery in Cluster 2 [50.0%], and no recovery in Cluster 1 and 3). However, no statistically significant lower risk was observed with Cluster 0 compared to the other phenotypes (RR: 0.70 [95% CI: 0.39–1.26], *P* = 0.24). Kaplan–Meier analysis of long-term hypertensive response stratified by phenotypes identified on k-means clustering is shown in Fig. 3C. Upon excluding patients with baseline HTN, Cluster 3 demonstrated a protective effect for hypertensive response to anthracycline chemotherapy compared to the other groups (HR: 0.30 [95% CI: 0.13– 0.67], *P* = 0.003). The relationship between systolic function (LVEF and GLS) and BP (SBP Z-score and DBP Z-score) and identified phenotypes over long-term follow-up was evaluated. GLS was significantly different between clusters (*P* = 0.017), while LVEF was not (*P* = 0.77) (Fig. 4A-B). The changes stratified by phenotypes identified on k-means clustering aligned with those observed using the primary outcome of CTRCD, with Cluster 1 demonstrating the most preserved GLS and LVEF. SBP Z-score was significantly different between clusters (*P* = 0.005), while DBP Z-score was not (*P* = 0.32) (Fig. 4C-D).

## Discussion

In this study, we applied unsupervised machine learning to a cohort of pediatric cancer patients undergoing anthracycline chemotherapy to identify and characterize distinct phenotypes. We observed four phenotypes characterized by diverse baseline clinical profiles, cardiac functional metrics, and longitudinal changes in blood pressure. These phenotypes exhibited varying susceptibility profiles to chemotherapy-related cardiotoxicity encompassing both CTRCD and hypertensive response. The growing application of cluster analyses in clinical research aims to reveal hidden structures within data, potentially overlooked by conventional observational or epidemiological studies [7]. This approach seeks to identify novel phenotypes linked to a disease or clinical syndrome [7]. 

**Fig. 5:**
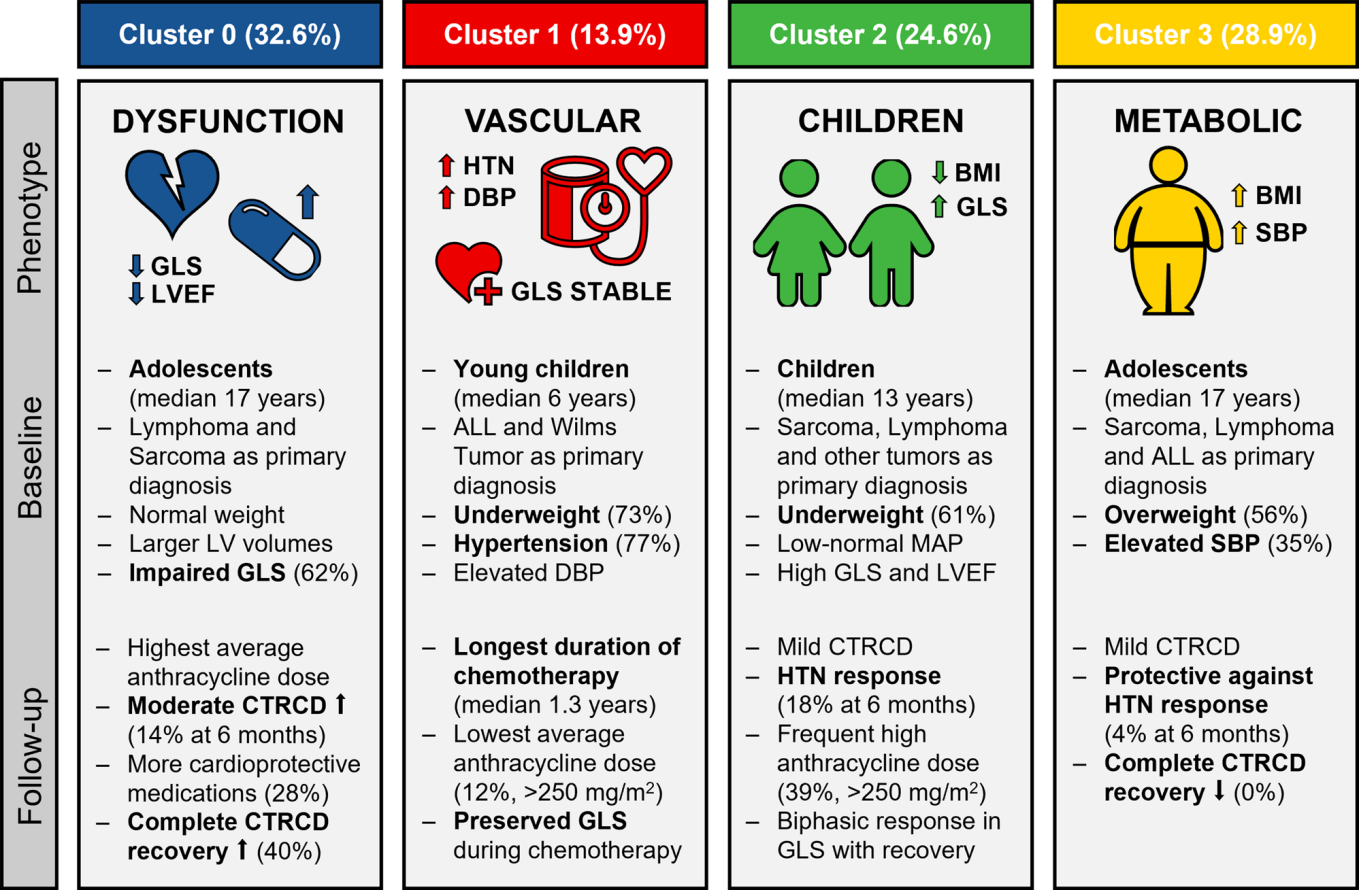
Visual summary of the distinct phengroups. Legend: Representation of the diverse clinical profiles, cardiac metrics, and adverse outcomes in the 4 identified clusters. ABBREVIATIONS: ALL, acute lymphoblastic leukemia; BMI, body mass index; CTRCD, cancer therapy-related cardiac dysfunction; DBP, diastolic blood pressure; GLS, global longitudinal strain; HTN, hypertension; LV, left ventricle; LVEF, left ventricular ejection fraction; MAP, mean arterial pressure; SBP, systolic blood pressure

Four distinct phenotypes were identified based on baseline characteristics which are visually summarized (Fig. 5). Cluster 0 comprised of 61 patients, representing predominantly male adolescents, with the most prevalent diagnosis within this cluster being lymphoma. Already at baseline this cluster demonstrated decreased systolic function, with the highest risk for moderate CTRCD during follow-up. Additionally, while the study revealed limited recovery from moderate CTRCD, Cluster 0 exhibited a higher trend towards complete recovery. Cluster 1, with only 26 patients, showed significantly younger age, were more likely underweight and hypertensive, with a higher prevalence of ALL and Wilms tumor as their primary diagnosis. The initial hypertension during acute presentation may be inherently linked to the primary diagnosis or, in part, attributed to induction chemotherapy, often involving high-dose corticosteroids [23, 24]. In both scenarios, hypertension frequently resolved shortly thereafter, such as in the case of Wilms tumor following nephrectomy and in ALL after the completion of induction chemotherapy [23, 24]. However, in both, an increased long-term risk of hypertension persisted [23, 24]. The blood pressure trajectory in Cluster 1 revealed a biphasic pattern, marked by initial surges, subsequent early recovery, and later steady increases during follow-up, which aligns with these findings. Another intriguing finding was the clustering of young patients with ALL in this particular group (mean age of 6.6 ± 3.5 years in Cluster 1 compared to 15.1 ± 5.4 years in the other clusters, *P* < 0.001), indicating that individuals of a younger age, previously proposed as a risk factor for increased hypertension incidence [24], may indeed constitute a distinct subgroup within the ALL population. Cluster 2, consisting of 46 patients with the highest frequency of female patients, had an intermediate age and a diverse distribution of diagnoses. At presentation, these patients were frequently underweight, and had the greatest cardiac function, with only a fraction demonstrating abnormal GLS. During follow-up, they frequently encountered declines in both GLS and LVEF resulting in mild CTRCD being common, while occurrences of moderate CTRCD were infrequent. Lastly, Cluster 3, comprising 54 patients, was characterized by excess body weight and obesity, and had high rates of mild CTRCD. A prevailing hypothesis suggests that obesity may trigger an upregulation of pro-inflammatory adipokines and a downregulation of anti-inflammatory adipokines, contributing to the development of a chronic, low-grade inflammatory state [25, 26]. This state may exacerbate the pro-inflammatory state induced by anthracyclines, potentially increasing susceptibility to cardiotoxicity. This may explain the occurrence of only CTRCD, and the concentration of these patients in this cluster, indicated by a clear visual separation on PC3. During follow-up these patients also frequently received angiotensin-converting enzyme inhibitors, with 7 out of 8 (87.5%) patients receiving angiotensin-converting enzyme inhibitors having experienced mild CTRCD, potentially indicating selection bias towards easier prescription in overweight patients with elevated SBP. The identification of distinct phenotypes carries significant implications for future clinical practice. For example, insights derived from our cluster phenotyping analysis could improve the detection of adverse events in prediction models by incorporating cluster membership as an additional predictor besides conventional risk stratification strategies. By extension, monitoring strategies and interventions could be subsequently based on patient cluster membership, leading to more effective prevention and management of CTRCD. The incorporation of machine learning algorithms into clinical practice is under investigation through a randomized feasibility trial at a single center, encompassing more than 200 adult cancer survivors [27]. These algorithms combine risk prediction with existing guidelines, expert consensus, and medical society scientific statements, potentially enhancing patient outcomes and reflecting a paradigm shift toward digital transformation in the cardiovascular care pathway [27]. 

### Study limitations

While acknowledging the strengths of our study, several limitations warrant consideration. First, the retrospective nature of the study introduces inherent biases and limits the ability to establish causation. Second, the limited sample size, while robustly characterized, may not fully capture the diversity of pediatric cancer patients undergoing chemotherapy on a worldwide scale. Furthermore, the reliance on echocardiography during routine follow-up may also overlook more subtle or intermittent cardiac changes. Lastly, due to the limited study duration these phenotypes may not fully capture differences in long-term sequelae, prompting the need for extended follow-up studies.

## Conclusions

Utilizing unsupervised machine learning algorithms, we identified four distinct phenotypes among pediatric patients with cancer treated with anthracycline chemotherapy. These phenotypes, characterized by diverse clinical profiles and cardiac metrics, displayed varying susceptibilities to adverse outcomes, highlighting the efficacy of machine learning algorithms in phenogrouping patients based on shared features and clinical progression. Ultimately, the integration of machine learning into clinical pediatric oncology practice holds promise for optimizing patient care.

## Electronic supplementary material

Below is the link to the electronic supplementary material.

Supplementary Material 1

## Data Availability

The data that support the findings of this study are not openly available due to privacy legislations and are available from the corresponding author upon reasonable request and approval from Johns Hopkins University.

## Abbreviations

CTRCD: Cancer therapy-related cardiac dysfunction
GLS: Global longitudinal strain
PCA: Principal component analysis
HTN: Hypertension
CDC: Centers for Disease Control and Prevention

## References

[1] Miller KD, Fidler-Benaoudia M, Keegan TH, Hipp HS, Jemal A, Siegel RL. Cancer statistics for adolescents and young adults, 2020. CA Cancer J Clin. 2020;70(6):443–59. 10.3322/caac.21637. 32940362 10.3322/caac.21637

[2] Chow EJ, Leger KJ, Bhatt NS, et al. Paediatric cardio-oncology: epidemiology, screening, prevention, and treatment. Cardiovasc Res. 2019;115(5):922–34. 10.1093/cvr/cvz031. 30768157 10.1093/cvr/cvz031 PMC6452306

[3] Getz KD, Sung L, Ky B, et al. Occurrence of treatment-related cardiotoxicity and its impact on outcomes among children treated in the AAML0531 clinical trial: a Report from the children’s Oncology Group. J Clin Oncol off J Am Soc Clin Oncol. 2019;37(1):12–21. 10.1200/JCO.18.00313. 10.1200/JCO.18.00313 PMC6354770 30379624

[4] Herrmann (Chair) J, Lenihan (Co-chair) Armenian D S, et al. Defining cardiovascular toxicities of cancer therapies: an International Cardio-Oncology Society (IC-OS) consensus statement. Eur Heart J. 2022;43(4):280–299. 10.1093/eurheartj/ehab674 10.1093/eurheartj/ehab674 PMC8803367 34904661

[5] Negishi T, Thavendiranathan P, Penicka M, et al. Cardioprotection using strain-guided management of potentially cardiotoxic Cancer therapy: 3-Year results of the SUCCOUR Trial. JACC Cardiovasc Imaging. 2023;16(3):269–78. 10.1016/j.jcmg.2022.10.010. 36435732 10.1016/j.jcmg.2022.10.010

[6] Kremer LCM, van der Pal HJH, Offringa M, van Dalen EC, Voûte PA. Frequency and risk factors of subclinical cardiotoxicity after anthracycline therapy in children: a systematic review. Ann Oncol off J Eur Soc Med Oncol. 2002;13(6):819–29. 10.1093/annonc/mdf167. 10.1093/annonc/mdf167 12123328

[7] Manlhiot C, van den Eynde J, Kutty S, Ross HJ. A primer on the Present State and Future prospects for machine learning and Artificial Intelligence Applications in Cardiology. Can J Cardiol. 2022;38(2):169–84. 10.1016/j.cjca.2021.11.009. 34838700 10.1016/j.cjca.2021.11.009

[8] Chaix MA, Parmar N, Kinnear C, et al. Machine learning identifies clinical and genetic factors Associated with Anthracycline Cardiotoxicity in Pediatric Cancer survivors. JACC CardioOncology. 2020;2(5):690–706. 10.1016/j.jaccao.2020.11.004. 34396283 10.1016/j.jaccao.2020.11.004 PMC8352204

[9] Feijen EAM, Leisenring WM, Stratton KL, et al. Derivation of Anthracycline and Anthraquinone Equivalence ratios to Doxorubicin for late-onset cardiotoxicity. JAMA Oncol. 2019;5(6):864–71. 10.1001/jamaoncol.2018.6634. 30703192 10.1001/jamaoncol.2018.6634 PMC6490232

[10] Kuczmarski RJ, Ogden CL, Guo SS, et al. CDC Growth Charts for the United States: methods and development. Vital Health Stat 11. 2000;2002(246):1–190. 12043359

[11] Flynn JT, Kaelber DC, Baker-Smith CM, et al. Clinical practice Guideline for Screening and Management of High Blood pressure in children and adolescents. Pediatrics. 2017;140(3). 10.1542/peds.2017-1904. 10.1542/peds.2017-1904 28827377

[12] Lai WW, Mertens LL, Cohen MS, Geva T. Echocardiography in Pediatric and congenital heart disease. 2nd ed. Wiley-Blackwell; 2016. 10.1002/9781118742440.

[13] Mitchell C, Rahko PS, Blauwet LA, et al. Guidelines for performing a comprehensive transthoracic echocardiographic examination in adults: recommendations from the American Society of Echocardiography. J Am Soc Echocardiogr. 2019;32(1):1–64. 10.1016/j.echo.2018.06.004. 30282592 10.1016/j.echo.2018.06.004

[14] Cohen JB, Brown NJ, Brown SA, et al. Cancer therapy–related hypertension: a Scientific Statement from the American Heart Association. Hypertension. 2023;80(3):e46–57. 10.1161/HYP.0000000000000224. 36621810 10.1161/HYP.0000000000000224 PMC10602651

[15] Chen T, Guestrin C, XGBoost:. A Scalable Tree Boosting System. In: Proceedings of the 22nd ACM SIGKDD International Conference on Knowledge Discovery and Data Mining. KDD ’16. Association for Computing Machinery. 2016;785–794. 10.1145/2939672.2939785

[16] Kursa MB, Rudnicki WR. Feature selection with the Boruta Package. J Stat Softw. 2010;036(i11).

[17] Bergstra J, Bengio Y. Random Search for Hyper-Parameter optimization. J Mach Learn Res. 2012;13:281–305.

[18] Snoek J, Larochelle H, Adams RP. Practical bayesian optimization of machine learning algorithms. In: Pereira F, Burges CJ, Bottou L, Weinberger KQ, editors. Advances in neural information Processing systems. Volume 25. Curran Associates, Inc.; 2012.

[19] Fouad KM, Ismail MM, Azar AT, Arafa MM. Advanced methods for missing values imputation based on similarity learning. PeerJ Comput Sci. 2021;7:e619. 10.7717/peerj-cs.619. 34395861 10.7717/peerj-cs.619 PMC8323724

[20] Ringnér M. What is principal component analysis? Nat Biotechnol. 2008;26(3):303–4. 10.1038/nbt0308-303. 18327243 10.1038/nbt0308-303

[21] Celebi ME, Kingravi HA, Vela PA. A comparative study of efficient initialization methods for the k-means clustering algorithm. Expert Syst Appl. 2013;40(1):200–10. 10.1016/j.eswa.2012.07.021.

[22] Arbelaitz O, Gurrutxaga I, Muguerza J, Pérez JM, Perona I. An extensive comparative study of cluster validity indices. Pattern Recognit. 2013;46:243–56.

[23] Hsiao W, Denburg M, Laskin B. Hypertension in Wilms tumor. Pediatr Nephrol. 2024;39(1):15–24. 10.1007/s00467-023-06011-y. 37178208 10.1007/s00467-023-06011-y PMC12281606

[24] Kumar R, Reed S, Stanek JR, Mahan JD. Defining kidney outcomes in children with acute lymphoblastic leukemia in the modern era. Pediatr Nephrol J Int Pediatr Nephrol Assoc. 2022;37(9):2119–26. 10.1007/s00467-021-05402-3. 10.1007/s00467-021-05402-3 35041040

[25] Pecoraro M, Del Pizzo M, Marzocco S, et al. Inflammatory mediators in a short-time mouse model of doxorubicin-induced cardiotoxicity. Toxicol Appl Pharmacol. 2016;293:44–52. 10.1016/j.taap.2016.01.006. 26780402 10.1016/j.taap.2016.01.006

[26] Guenancia C, Lefebvre A, Cardinale D, et al. Obesity as a risk factor for anthracyclines and trastuzumab cardiotoxicity in breast Cancer: a systematic review and Meta-analysis. J Clin Oncol off J Am Soc Clin Oncol. 2016;34(26):3157–65. 10.1200/JCO.2016.67.4846. 10.1200/JCO.2016.67.4846 PMC5569689 27458291

[27] Brown SA, Chung BY, Doshi K, et al. Patient similarity and other artificial intelligence machine learning algorithms in clinical decision aid for shared decision-making in the Prevention of Cardiovascular toxicity (PACT): a feasibility trial design. Cardio-oncology. 2023;9(1):7. 10.1186/s40959-022-00151-0. 36691060 10.1186/s40959-022-00151-0 PMC9869606

