# Supplementary material for: Unsupervised machine learning identifies distinct phenotypes in cardiac complications of pediatric patients treated with anthracyclines
Source: Cardiooncology. 2024 Oct 28;10:74. doi: 10.1186/s40959-024-00276-4 (PMC11514752; doi:10.1186/s40959-024-00276-4)
Supplement: Supplementary file 1 — Supplementary Material 1 [file 40959_2024_276_MOESM1_ESM.docx]

**Supplemental Table 1.** Feature variables included in principal component analysis**.**

| **Variables** | **Features considered** |
| --- | --- |
| Demographics |  |
| Age, years | 1 |
| Male, n (%) | 1 |
| Diagnosis | 7 |
|  |  |
| Clinical assessment |  |
| BSA, m^2^ |  |
| BMI Z-score | 1 |
| Height Z-score | 1 |
| Weight Z-score | 1 |
| Systolic BP Z-score | 3^*^ |
| Diastolic BP Z-score | 3^*^ |
| MAP Z-score | 1 |
| HTN classification |  |
| Stage 1 HTN, n (%) | 1 |
| Stage 2 HTN, n (%) | 1 |
|  |  |
| Echocardiography assessment |  |
| LVESVi, ml/m^2^ | 1 |
| LVEDVi, ml/m^2^ | 1 |
| LVFS, % | 3^*^ |
| LVEF, % | 3^*^ |
| LVEF_3D_, % | 3^*^ |
| GLS, % | 3^*^ |
| TAPSE, cm | 3^*^ |
|  |  |
| Follow-up |  |
| Duration of chemotherapy, years | 1 |
| Cumulative anthracycline exposure^a^, mg/m^2^ | 1 |

LEGEND: Feature variables were obtained using a quadratic function according to the following formula: (Variable)_T0_+Slope*Time+Quadratic Coefficient*Time, and 3 variables were extracted per patient (Intercept, Slope and Quadratic Coefficient. ABBREVIATIONS: ALL, acute lymphoblastic leukemia; AML, acute myeloid leukemia; BMI, body mass index; GLS, global longitudinal strain; HTN, hypertension; LVEF, left ventricular ejection fraction, LVEDV, LV end diastolic volume, LVESV, LV end systolic volume, LVFS, left ventricular fractional shortening; LVEDVi, LVEDV indexed to BSA; LVESVi, LVESV indexed to BSA; MAP, mean arterial pressure; TAPSE, tricuspid annular plane systolic excursion.

**Supplemental Table 2.** Eigenanalysis of the Correlation Matrix

| Principal Component | Explained Variance Ratio | Eigenvalue | Cumulative Variance Ratio |
| --- | --- | --- | --- |
| PC1 | 0.129 | 5.264 | 0.129 |
| PC2 | 0.12 | 4.809 | 0.249 |
| PC3 | 0.105 | 4.19 | 0.354 |
| PC4 | 0.068 | 2.771 | 0.422 |
| PC5 | 0.056 | 2.297 | 0.478 |
| PC6 | 0.055 | 2.236 | 0.533 |
| PC7 | 0.05 | 2.022 | 0.583 |
| PC8 | 0.046 | 1.892 | 0.629 |
| PC9 | 0.041 | 1.841 | 0.67 |
| PC10 | 0.037 | 1.515 | 0.707 |
| PC11 | 0.035 | 1.436 | 0.742 |
| PC12 | 0.03 | 1.234 | 0.773 |
| PC13 | 0.027 | 1.101 | 0.8 |
| PC14 | 0.025 | 1.02 | 0.825 |
| PC15 | 0.022 | 0.892 | 0.847 |
| PC16 | 0.019 | 0.77 | 0.866 |
| PC17 | 0.017 | 0.691 | 0.882 |
| PC18 | 0.016 | 0.659 | 0.899 |
| PC19 | 0.016 | 0.636 | 0.914 |
| PC20 | 0.011 | 0.465 | 0.926 |
| PC21 | 0.009 | 0.368 | 0.935 |
| PC22 | 0.008 | 0.341 | 0.943 |
| PC23 | 0.007 | 0.305 | 0.951 |
| PC24 | 0.006 | 0.234 | 0.956 |
| PC25 | 0.005 | 0.203 | 0.961 |
| PC26 | 0.004 | 0.18 | 0.966 |
| PC27 | 0.004 | 0.172 | 0.97 |
| PC28 | 0.004 | 0.149 | 0.974 |
| PC29 | 0.003 | 0.128 | 0.977 |
| PC30 | 0.003 | 0.113 | 0.979 |
| PC31 | 0.003 | 0.112 | 0.982 |
| PC32 | 0.003 | 0.102 | 0.985 |
| PC33 | 0.002 | 0.086 | 0.987 |
| PC34 | 0.002 | 0.078 | 0.989 |
| PC35 | 0.002 | 0.073 | 0.99 |
| PC36 | 0.001 | 0.058 | 0.992 |
| PC37 | 0.001 | 0.057 | 0.993 |
| PC38 | 0.001 | 0.049 | 0.994 |
| PC39 | 0.001 | 0.042 | 0.996 |
| PC40 | 0.001 | 0.038 | 0.996 |
| PC41 | 0.001 | 0.035 | 0.997 |
| PC42 | 0.001 | 0.035 | 0.998 |
| PC43 | 0.001 | 0.033 | 0.999 |
| PC44 | 0.001 | 0.021 | 0.999 |
| PC45 | 0 | 0.02 | 1 |
| PC46 | 0 | 0 | 1 |
| PC47 | 0 | 0 | 1 |

**Supplemental Table 3.** Eigenvectors of the first 5 principal components.

| **Variables** | **PC1** | **PC2** | **PC3** | **PC4** | **PC5** |
| --- | --- | --- | --- | --- | --- |
| Duration of chemotherapy, years | 0.037 | 0.056 | 0.044 | -0.14 | 0.121 |
| Cumulative anthracycline exposure | -0.118 | -0.075 | -0.004 | -0.029 | -0.008 |
| Age, years | -0.082 | -0.195 | 0.182 | 0.08 | 0.028 |
| Body surface area | -0.047 | -0.267 | 0.264 | 0.023 | -0.016 |
| Systolic BP Z-score | 0.314 | -0.071 | 0.155 | -0.084 | 0.109 |
| Diastolic BP Z-score | 0.357 | 0.014 | 0.008 | -0.11 | 0.045 |
| MAP Z-score | 0.379 | -0.03 | 0.084 | -0.11 | 0.082 |
| LVFS, % | -0.038 | 0.242 | 0.211 | 0.087 | -0.124 |
| GLS, % | -0.011 | -0.297 | -0.07 | 0.044 | -0.238 |
| LVEF, % | -0.026 | 0.273 | 0.173 | -0.027 | 0.102 |
| TAPSE, cm | -0.051 | -0.077 | 0.246 | 0.093 | 0.304 |
| LVESVi, ml/m^2^ | -0.001 | -0.258 | -0.131 | 0.155 | 0.024 |
| LVEDVi, ml/m^2^ | -0.022 | -0.148 | -0.033 | 0.178 | 0.103 |
| SBP Z-score (intercept) | 0.287 | -0.096 | 0.183 | -0.071 | 0.095 |
| SBP Z-score (quadratic coefficient) | 0.202 | 0.077 | -0.022 | 0.167 | -0.196 |
| SBP Z-score (slope) | -0.304 | -0.047 | 0.001 | -0.159 | 0.069 |
| DBP Z-score (intercept) | 0.361 | -0.003 | 0.027 | -0.113 | 0.028 |
| DBP Z-score (quadratic coefficient) | 0.221 | 0.057 | -0.109 | 0.254 | -0.03 |
| DBP Z-score (slope) | -0.325 | -0.04 | 0.054 | -0.171 | -0.001 |
| LVFS, % (intercept) | -0.027 | 0.277 | 0.183 | 0.067 | -0.096 |
| LVFS, % (quadratic coefficient) | 0.06 | 0.165 | 0.068 | 0.076 | -0.202 |
| LVFS, % (slope) | 0.002 | -0.197 | -0.128 | -0.062 | 0.208 |
| LVEF, % (intercept) | -0.007 | 0.279 | 0.159 | -0.039 | 0.14 |
| LVEF, % (quadratic coefficient) | -0.082 | 0.003 | 0.151 | -0.234 | -0.118 |
| LVEF, % (slope) | 0.108 | -0.078 | -0.179 | 0.201 | -0.004 |
| LVEF_3D_, % (intercept) | 0.058 | 0.036 | 0.028 | 0.085 | 0.268 |
| LVEF_3D_, % (quadratic coefficient) | 0.042 | -0.048 | -0.091 | 0.264 | 0.263 |
| LVEF_3D_, % (slope) | -0.018 | 0.077 | 0.059 | -0.276 | -0.316 |
| GLS, % (intercept) | -0.009 | -0.31 | -0.059 | -0.016 | -0.225 |
| GLS, % (quadratic coefficient) | -0.07 | -0.116 | -0.054 | 0.1 | 0.06 |
| GLS, % (slope) | -0.025 | 0.223 | 0.116 | -0.022 | -0.011 |
| TAPSE, cm (intercept) | -0.07 | -0.071 | 0.295 | 0.12 | 0.311 |
| TAPSE, cm (quadratic coefficient) | -0.111 | -0.017 | 0.064 | 0.029 | 0.14 |
| TAPSE, cm (slope) | 0.139 | 0.004 | -0.175 | -0.028 | -0.234 |
| Male, n (%) | 0.024 | -0.036 | 0.028 | 0.03 | 0.024 |
| Stage 1 HTN, n (%) | 0.053 | 0.01 | -0.014 | -0.01 | 0.006 |
| Stage 2 HTN, n (%) | 0.099 | 0.03 | -0.023 | -0.008 | 0.016 |
| Lymphoma, n (%) | -0.028 | -0.039 | 0.008 | -0.014 | -0.032 |
| Sarcoma, n (%) | -0.007 | -0.026 | 0.007 | 0.013 | -0.014 |
| AML, n (%) | -0.016 | -0.005 | -0.006 | -0.002 | -0.005 |
| ALL, n (%) | 0.026 | 0.008 | 0.018 | 0.008 | 0.033 |
| Blastoma, n (%) | 0.016 | 0.028 | -0.017 | 0.005 | -0.009 |
| Wilms tumor, n (%) | 0.012 | 0.018 | -0.019 | -0.011 | 0.011 |
| Other, n (%) | -0.003 | 0.017 | 0.009 | 0.001 | 0.016 |
| Weight Z-score | 0.077 | -0.243 | 0.393 | -0.047 | -0.16 |
| Height Z-score | 0.087 | -0.211 | 0.015 | -0.563 | 0.125 |
| BMI Z-score | 0.018 | -0.166 | 0.459 | 0.301 | -0.295 |

**Supplemental Figure 1. Scree plot**


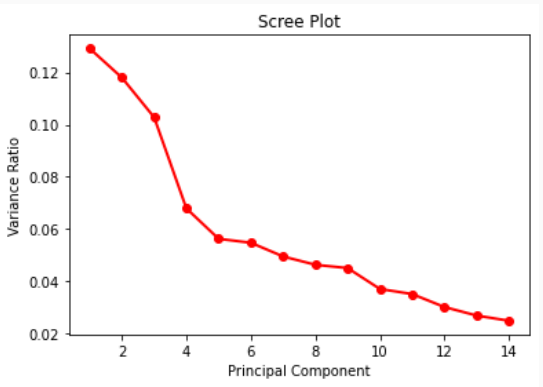


The scree plot displays the variance ratio explained by each principal component (PC), with the first 14 PCs shown. The x-axis denotes the PCs, and the y-axis shows the variance ratio. Red dots represent each PC, connected by a line to illustrate the trend. The "elbow" at PC = 5 indicates the optimal cutoff where additional components contribute minimal additional variance. This heuristic guides the decision to retain the first 5 PCs, balancing variance capture with model simplicity for subsequent analysis, including K-means clustering.

**Supplemental Figure 2. Three-dimensional loading plots**

**
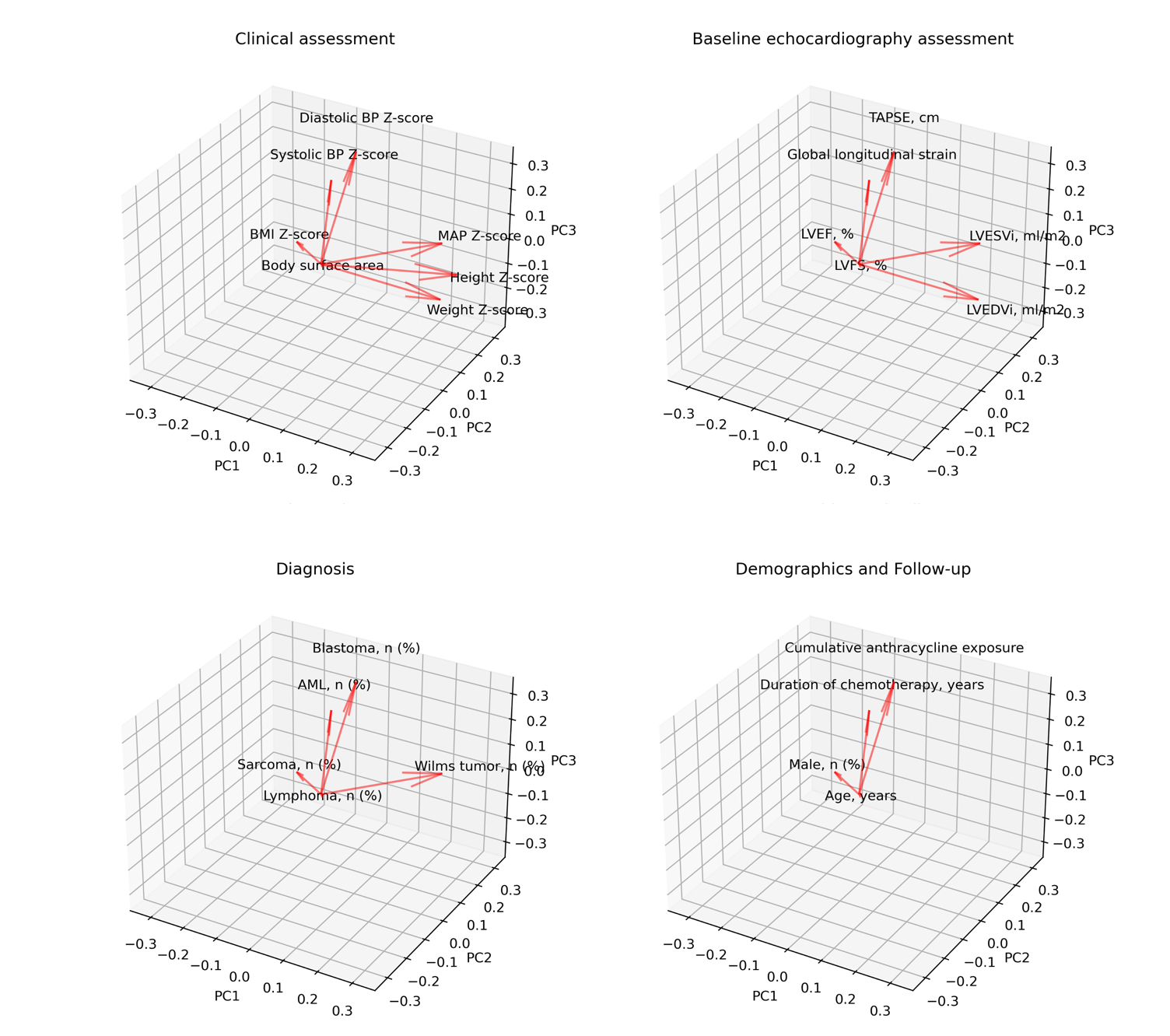
**

3D plot displaying the loadings of each feature on the first three principal components (PC1, PC2 and PC3) with arrows indicating the directions of the features. The length and direction of the arrows reflect the magnitude and sign of each feature’s influence on the principal components.
